# Validation and clinical utility of OMGRate and MG-QOL15 in evaluating symptoms and quality of life in Chinese patients with ocular myasthenia gravis

**DOI:** 10.3389/fneur.2026.1842388

**Published:** 2026-05-22

**Authors:** Ting Tang, Qiwen Feng, Youran Cai, Yuying Dong, Jian Chen, Rijia Zhang, Qing Zhou

**Affiliations:** 1 Department of Ophthalmology, The First Affiliated Hospital of Jinan University, Guangzhou, China; 2 Department of Ophthalmology, Dongguan Binhaiwan Central Hospital, Dongguan, China

**Keywords:** MG-QOL15 scales, ocular myasthenia gravis, OMGRate scale, quality of life, validate

## Abstract

**Purpose:**

This study aimed to evaluate the value of the OMGRate and MG-QOL 15 in assessing quality of life (QoL) before and after treatment in ocular myasthenia gravis (OMG) and to validate the applicability of OMGRate in the Chinese OMG population.

**Methods:**

This prospective observational cohort study was conducted via questionnaire among patients diagnosed with OMG at our hospital between November 2022 and January 2024. Patient outcomes were assessed at pre-treatment (baseline), 1, 3, and 6 months post-treatment. Efficacy was assessed using the OMGRate and O-QMGS, while the MG-QOL15 was used to evaluate the QoL of OMG patients.

**Results:**

Our study included 52 patients with OMG. Age of onset and first Symptoms significantly impacted patients’ QoL (*p* < 0.001); AChR-Ab positivity and thymic abnormalities were associated with disease severity (*p* < 0.05). Longitudinal observations revealed significant reductions in all scale scores (OMGRate, MG-QOL15, O-QMGS), reaching optimal levels at 6 months. OMGRate-e and OMGRate-q scores demonstrated moderate to strong positive correlations across all observation times; OMGRate and OMGRate-q scores showed positive correlations with MG-QOL15 scores at all observation time points; OMGRate and OMGRate-e scores showed positive correlations with O-QMGS scores only at post-treatment observation time points.

**Conclusion:**

The OMGRate was validated as an effective method for severity assessment in Chinese OMG patients. OMG significantly impairs patients’ QoL, whereas standardised treatment not only effectively alleviates clinical symptoms but also concurrently improves quality of life, revealing good consistency between symptomatic improvement and patient-reported outcomes.

## Background

Ocular Myasthenia Gravis (OMG) is an autoimmune disorder primarily characterized by isolated involvement of the extraocular muscles (1, 2), affecting the extraocular muscles, levator palpebrae superioris and orbicularis oculi muscles. Clinical manifestations include ptosis, diplopia/strabismus (3–6) and lagophthalmos (6), and these symptoms impair patients’ quality of life (QoL) severely (7). OMG patients showed ocular symptoms only at the beginning of this disease, while approximately 39% of patients carry a risk of progression to Generalized Myasthenia Gravis (GMG) (8), this trend of transformation particularly occurs in the early stages of OMG (2). Due to extraocular muscle involvement, OMG patients manifested as visual development impairment as well as diminished daily functional abilities, such as difficulty in eye opening, reading, and driving. Furthermore, OMG induces significant social anxiety and psychological distress and then influences overall QoL in the disease course (9).

However, existing myasthenia gravis (MG) assessment scales were initially devised for evaluating GMG (10) and appear to have insufficient sensitivity and specificity in OMG patients. They are inadequate in assessing the OMG symptoms and the impact of ocular manifestations on patients’ daily lives; therefore, it is essential to formulate an OMG-oriented scale to quantify disease severity more accurately. Its effect on QoL remains a critical and unresolved clinical challenge. To assess the symptom burden and QoL in OMG patients thoroughly, Sui Hsien Wong et al. (11) developed the ocular myasthenia gravis rating scale (OMGRate) and validated its reliability and validity in 2019. The OMGRate consists of two components: a physician-examination (OMGRate-e) and a patient questionnaire (OMGRate-q). Subsequent multicenter studies have further confirmed OMGRate-q as a valid and reliable disease-specific patient-reported outcome measure (PROM) (12) by providing targeted assessment of symptoms of quantity ptosis and diplopia and sensitively dynamic changes in disease severity. Additionally, the myasthenia gravis quality of life 15-item scale (MG-QOL15), an internationally recognized tool for assessing QoL in MG, has a comprehensive assessment covering 15 psychological and physiological dimension indicators, which can systematically evaluate the disease’s impact on patients’ social functioning, emotional status, and daily activities (13–15).

This study aimed to integrate the OMGRate and MG-QOL15 to quantify symptom severity and elaborate on the comprehensive impact of OMG on QoL in multiple dimensions, and validate the applicability of OMGRate in the Chinese OMG population, thereby providing a more robust proof for clinical decision-making and efficacy assessment.

## Methods

### Study subjects

**Figure 1:**
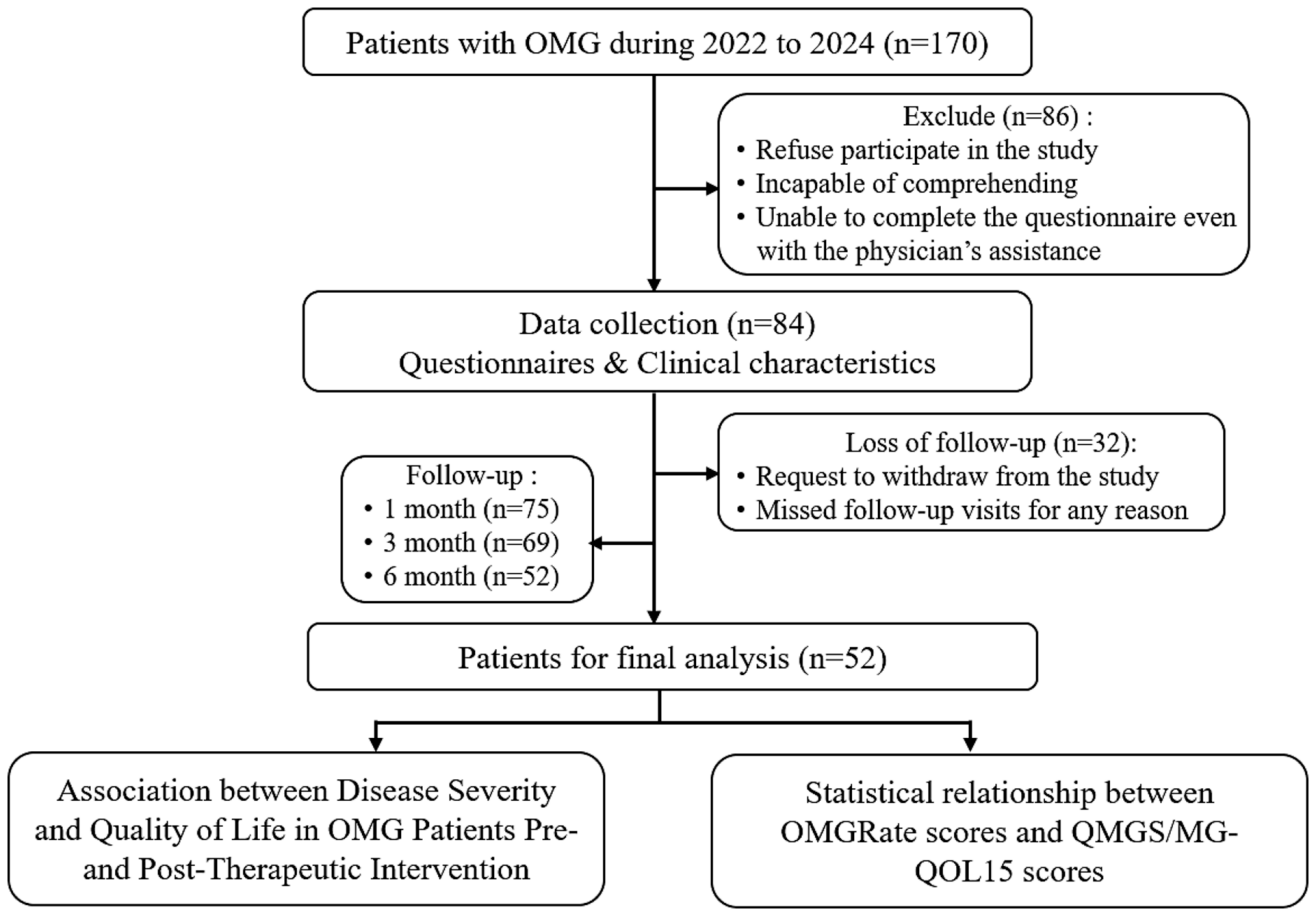
The flowchart of the research design.

This was a prospective observational cohort study. We enrolled patients diagnosed with OMG in the Department of Ophthalmology at the First Affiliated Hospital of Jinan University between November 2022 and January 2024. Participants were aged ≥10 years and had not received treatment before this study. Our diagnosis of OMG was based on the Chinese guidelines for the diagnosis and treatment of myasthenia gravis(2020 version) (6): the presence of fluctuating ocular muscle weakness and a positive result in at least one of the following tests. Tests include serological antibodies assay [acetylcholine receptor antibody (AChR-Ab), muscle-specific kinase antibody (MuSK-Ab), low-density lipoprotein receptor 4 antibody (LRP 4-Ab)], electrophysiological examination (repetitive nerve stimulation [RNS] or singlefiber electromyography [SFEMG]), or a neostigmine test. Patients with ocular palsies of other etiologies or those with GMG were excluded. All participants were required to complete a follow-up of at least 6 months. Our study protocol was approved by the First Affiliated Hospital of Jinan University Ethics Committee (Approval No.: KY-2021-031). The overall workflow of the study is illustrated in Figure 1.

### Inclusion criteria and exclusion criteria

Inclusion criteria for our study: 1) patients initially diagnosed with OMG at our hospital’s ophthalmology outpatient clinic; 2) a follow-up duration of no less than 6 months after diagnosis. Exclusion criteria include: 1) concomitant other diseases, such as congenital ptosis, Sjögren’s syndrome, etc.; 2) patients progress to GMG during the follow-up period; 3) patients unable to complete the patient-reported questionnaires (OMGRate-q and MG-QOL15) even with the physician’s assistance; 4) previous treatment with immunosuppressive drugs, targeted biologics, etc.; and 5) history of strabismus surgery or thymectomy.

### Grouping

Study participants were stratified by age of onset into the following groups: juvenile ocular myasthenia gravis (JOMG, ≥10 and <18 years), Adult OMG, which was further subdivided into early-onset ocular myasthenia gravis (EOMG, ≥18 and ≤50 years), and late-onset ocular myasthenia gravis (LOMG, >50 years). Subgroup analysis based on predominant symptoms at enrollment categorized patients according to the first symptom into the ptosis group, the diplopia/strabismus group, and the group with combined ptosis and diplopia/strabismus.

### Therapeutic strategy

The therapeutic strategy in our study was based on the Chinese guidelines for the diagnosis and treatment of myasthenia gravis (2020 version) (6), which recommends acetylcholinesterase inhibitors (such as pyridostigmine bromide) as the first-line treatment for symptom relief. Immunosuppressive drugs (e.g., prednisone) were used for those OMG patients whose symptoms proved difficult to control or severely impacted QoL, following the principle of “low-dose incremental method”.

### Data collection

At the initial visit, a comprehensive dataset was collected, including patient demographics (sex, age, age of onset, first symptoms) and clinical results from examinations such as ptosis and extraocular motility examination, Red-Glass Test, ice pack test, neostigmine test, serum AChR-Ab test, thyroid function, immune function, RNS, chest CT, and thyroid ultrasound. Thyroid color Doppler ultrasound results indicating abnormal thyroid morphology and size, and thyroid function tests showing TSH, FT3, or FT4 levels beyond the normal reference range, are recorded as thyroid abnormalities. Chest CT suggesting thymic hyperplasia or thymoma is recorded as thymus abnormal. Deviations of lymphocyte counts, immunoglobulin, or complement levels from the normal range are recorded as an immune abnormality. The physician performed face-to-face assessments using the OMGRate-e (11) and Ocular Quantitative Myasthenia Gravis Score (O-QMGS) (16) scales. Patients self-completed the OMGRate-q (11) and MG-QOL15 (13) with the physician’s guidance (Explain medical terms that patients cannot understand, or read entries for those with reading difficulties). Treatment response study evaluation was conducted at fixed observation points (pre-treatment [baseline], 1, 3, and 6 months post-treatment) using OMGRate (OMGRate-e & OMGRate-q), O-QMGS, and MG-QOL15. Record scores of these scales at each follow-up.

### Translation and cross-cultural adaptation of the OMGRate

The original version of OMGRate (OMGRate-e & OMGRate-q) was translated into a Chinese version and cross-culturally adapted according to published international guidelines (17, 18). The research committee, comprising five ophthalmology specialists and one linguistics expert, was recruited. Using the forward translation method outlined in the guidelines, OMGRate was translated into Chinese, then reverse-translated back into the original version. The aforementioned committee reviewed both the forward and back-translated versions of OMGRate, discussed discrepancies, and produced a consolidated bilingual translation. By analysing all translated wording and comparing it with the original text, a final version of the OMGRate scale was developed to achieve equivalent validity with the original version. Regarding cognitive function assessment, twenty OMG patients participated in a pilot study utilising the latest Chinese version of OMGRate. The final Chinese version of OMGRate was subsequently produced. Finally, all members reached consensus regarding the equivalence of the final Chinese version to the original English version.

### Data analysis

All data were analyzed statistically using SPSS 26.0 and were visualized using GraphPad Prism 8.0. Continuous data were presented as mean ± standard deviation (SD) or median [interquartile range (IQR)], while categorical data were expressed as frequencies (percentages). Continuous variables were assessed for normality using the Shapiro–Wilk test. As the data were non-normally distributed, intergroup comparisons employed the Kruskal-Wallis H test or Mann–Whitney U test to analyze the impact of demographic factors and clinical characteristics. The Friedman test was used to analyze scale scores (OMGRate, OMGRate-q, OMGRate-e, MG-QOL15, O-QMGS) across different follow-up time points (pre-treatment/baseline and post-treatment). Spearman Correlation Analysis was used to assess the correlations among the OMGRate (OMGRate-q, OMGRate-e), MG-QOL15, and O-QMGS scores before and after treatment. Cronbach’s alpha coefficients was calculated for OMGRate, OMGRate-e, and OMGRate-q as part of a post-hoc analysis of internal consistency. Post-hoc comparisons conducted using the Bonferroni-corrected method. *p* < 0.05 was considered statistically significant.

## Results

After assessment based on the criteria, 86 patients were excluded, and 32 were lost to follow-up; a total of 52 patients were included in this study (Figure 1). Of all the participants 19(36.5%) were men, and 33(63.5%) were women. The mean age of onset was 34.0 ± 19.1 years. Detailed demographic, clinical characteristics, and therapeutic strategy of participants are presented in Supplementary Table S1.

### Clinical association between OMGRate and MG-QOL15 scores

As displayed in Table 1, the OMGRate scores for the JOMG, EOMG, and LOMG groups were 6.00 (3.00, 19.00), 32.50 (8.75, 43.00), and 37.00 (11.00, 50.00), respectively; there were statistically significant differences (*p* = 0.027). However, pairwise comparisons were not statistically significant. In contrast, the MG-QOL15 scores of the three groups were 6.00 (1.50, 12.00), 21.00 (9.25, 31.25), and 32.00 (30.00, 37.00), respectively, demonstrating a notable significant difference (*p* < 0.001). Furthermore, the pairwise comparisons among all groups for MG-QOL15 scores were statistically significant.

**Table 1:** Analysis of factors affecting pre-treatment OMGRate and MG-QOL15 scores in OMG patients based on clinical characteristics.

Characteristic	Category	Number (%)	OMGRate scores	*Z/H* value	*p*-value	MG-QOL15 scores	*Z/H* value	*p*-value
Sex	Male	19 (36.5%)	22.00 (5.00, 46.00)	−0.114	0.909	9.00 (1.00, 30.00)	−1.626	0.104
	Female	33 (63.5%)	25.00 (7.00, 40.50)			24.00 (11.00, 32.00)		
Age of Onset	JOMG	13 (25%)	6.00 (3.00, 19.00)	7.249	0.027^*^	6.00 (1.50, 12.00) ^a^	19.085	<0.001^***^
	EOMG	28 (53.8%)	32.50 (8.75, 43.00)			21.00 (9.25, 31.25)^a^		
	LOMG	11 (21.2%)	37.00 (11.00, 50.00)			32.00 (30.00, 37.00) ^a^		
First Symptoms	Ptosis only	12 (23.2%)	4.00 (2.25, 6.75)	18.347	<0.001 ^***^	9.00 (2.75, 25.50)	3.745	0.154
	Diplopia/strabismus	25 (48.1%)	37.00 (17.00, 44.50) ^b^			24.00 (11.00, 32.00)		
	Ptosis with diplopia/strabismus	15 (28.8%)	36.00 (11.00, 49.00) ^b^			30.00 (4.00, 39.00)		
Serum AChR-Ab test	Positive	6 (12.5%)	38.50 (20.00, 52.25)	−1.747	0.081	34.50 (24.50, 46.25)	−2.247	0.025^*^
	Negative	42 (87.5%)	21.00 (5.00, 42.25)			19.00 (5.75, 30.00)		
Thyroid	Abnormal	31 (64.6%)	25.00 (7.00, 43.00)	−0.356	0.722	21.00 (8.00, 30.00)	−0.313	0.754
	Normal	17 (35.4%)	31.00 (5.50, 41.50)			17.00 (5.50, 34.50)		
Thymus	Abnormal	21 (42.9%)	14.00 (5.50, 25.50)	−2.547	0.011^*^	20.00 (2.50, 28.00)	−1.223	0.221
	Normal	28 (57.1%)	37.50 (11.00, 45.25)			26.00 (8.25, 32.00)		

*p < 0.05, **p < 0.01, ***p < 0.001. a represents the pairwise comparison of the age of onset was statistically significant. b represents compared with ptosis, other first symptoms were statistically significant. ^#^Among the AChR-Ab negative patients, the diagnosis of OMG was confirmed by RNS in 3 patients (7.14%), positive neostigmine test in 36 patients (85.72%), and symptoms significantly relieved after treatment in 3 patient (7.14%).

A statistically significant difference in OMGRate scores was observed among the groups stratified by the first symptom (*p* < 0.001). The median OMGRate score in the ptosis group (4.00; IQR: 2.25–6.75) was lower than that in both the diplopia/strabismus group (37.00; IQR: 17.00–44.50) and the combined ptosis with diplopia/strabismus group (36.00; IQR: 11.00–49.00). There were significant pairwise differences between the ptosis group and the diplopia/strabismus group (*p* < 0.001), also between the ptosis group and the combined symptoms group (*p* = 0.001). However, the difference in MG-QOL15 scores across these symptom-based groups was not statistically significant (*p* = 0.154).

The OMGRate scores of the AChR-Ab positive group (38.50 [20.00–52.25]) were higher than the AChR-Ab negative group [21.00 (5.00–42.25)] (*p* = 0.081). Conversely, the MG-QOL15 scores were significantly higher in the AChR-Ab positive group (34.50 [24.50–46.25]) than in the negative group [19.00 (5.75–30.00)] (*p* = 0.025). Patients with thymic abnormalities had lower OMGRate scores (14.00 [5.50–25.50]) than those with a normal thymus [37.50 (11.00–45.25)], (*p* = 0.011). However, the MG-QOL15 scores showed no significant difference. Furthermore, no statistically significant differences were shown in gender or thyroid function status (*p* > 0.05).

### Dynamic changes in OMGRate, MG-QOL15, and O-QMGS scores before and after therapeutic intervention

As shown in Table 2 and Supplementary Figures S1–S3, all scores (OMGRate, OMGRate-e, OMGRate-q, MG-QOL15, and O-QMGS) presented a remarkable reduction over the course of therapeutic intervention. A statistically significant reduction in scores was observed at the 3-month follow-up compared with baseline. At the 6-month follow-up, all scales’ scores had reached their lowest in our observation period.

**Table 2:** OMGRate, MG-QOL15, and O-QMGS scores of patients at different follow-up times.

Scales	Baseline	1-month follow-up	3-month follow-up	6-month follow-up	*χ^2^*	*p*-value
OMGRate	24.50 (6.25, 42.75)	10.50 (3.00, 39.50)	6.00 (3.00, 28.00)	4.00 (1.00, 19.00)	30.669	<0.001
OMGRate-q	21.00 (5.25, 34.75)	9.00 (3.00 ~ 32.50)	4.50 (3.00, 22.50)	4.00 (1.00, 15.75)	28.394	<0.001
OMGRate-e	5.00 (0.00, 8.00)	2.00 (0.00, 8.00)	0.00 (0.00 ~ 8.00)	0.00 (0.00, 3.75)	50.258	<0.001
MG-QOL15	21.00(8.00, 31.50)	12.00(4.25, 26.00)	12.50(3.00, 20.00)	5.50(0.25, 17.00)	22.799	<0.001
O-QMGS	3.00 (3.00, 3.00)	3.00 (3.00, 3.00)	3.00 (0.00, 3.00)	3.00 (0.00, 3.00)	46.149	<0.001

The Friedman test was used to compare scores across different follow-up time points.

At baseline, the OMGRate median scores were 24.50 (IQR: 6.25–42.75); OMGRate-q were 21.00 (IQR: 5.25–34.75), OMGRate-e were 5.00 (IQR: 0.00–8.00); MG-QOL15 were 21.00 (IQR: 8.00–31.50), and O-QMGS were 3.00 (IQR: 3.00–3.00). After 6 months of treatment, the median scores reduced, with OMGRate 4.00 (IQR: 1.00–19.00), OMGRate-q 4.00 (IQR: 1.00–15.75), OMGRate-e 0.00 (IQR: 0.00–3.75), MG-QOL15 5.50 (IQR: 0.25–17.00), and O-QMGS 3.00 (IQR: 0.00–3.00).

### Longitudinal changes in disease symptom severity and HRQOL

The OMGRate and O-QMGS were used to assess disease severity, while the MG-QOL15 was used to evaluate health-related quality of life (HRQOL).

In our study, both the OMGRate-q and MG-QOL15 scores presented a reducing trend after treatment, with largely consistent trajectories (Table 2, Supplementary Figure S1). The median OMGRate-q score decreased from 21.00 (IQR: 5.25–34.75) at baseline to 4.00 (IQR: 1.00–15.75) after 6 months of treatment. Similarly, the median MG-QOL15 score reduced from 21.00 (IQR: 8.00–31.50) to 5.50 (IQR: 0.25–17.00) within the same interval (Table 2). Furthermore, while scores on both scales were widely dispersed at baseline, the IQR substantially narrowed after therapeutic intervention.

Both the OMGRate-e and O-QMGS scores showed a reducing trend, which is consistent with symptom relief and QoL improvement. The range of reduction was more prominently pronounced for OMGRate-e than for O-QMGS. The O-QMGS scores showed minimal variation over time (Table 2, Supplementary Figures S2, S3), indicating that this scale may lack the sensitivity to precisely represent clinical changes in disease status throughout the treatment course.

### Reliability and validity of OMGRate

The internal consistency of OMGRate was evaluated using Cronbach‘s *α* coefficient at baseline and at each follow-up time point. As shown in Table 3, the OMGRate-q demonstrated good to excellent internal consistency, with Cronbach’s α values ranging from 0.875 at baseline to 0.923 at 6 months. The OMGRate-e also showed acceptable to good internal consistency, with α coefficients ranging from 0.805 at baseline to 0.868 at 3 months. The total OMGRate exhibited excellent internal consistency across all time points, with α values ranging from 0.891 at baseline to 0.931 at 6 months. These results indicate that OMGRate, and its subscales, has satisfactory internal consistency reliability in assessing symptom severity in Chinese patients with ocular myasthenia gravis.

**Table 3:** Internal consistency of OMGRate.

Scales	Items	Cronbach’s α coefficient			
		Baseline	1-month follow-up	3-month follow-up	6-month follow-up
OMGRate-q	11	0.875	0.909	0.899	0.923
OMGRate-e	6	0.805	0.850	0.868	0.850
OMGRate	17	0.891	0.923	0.917	0.931

**Figure 2:**
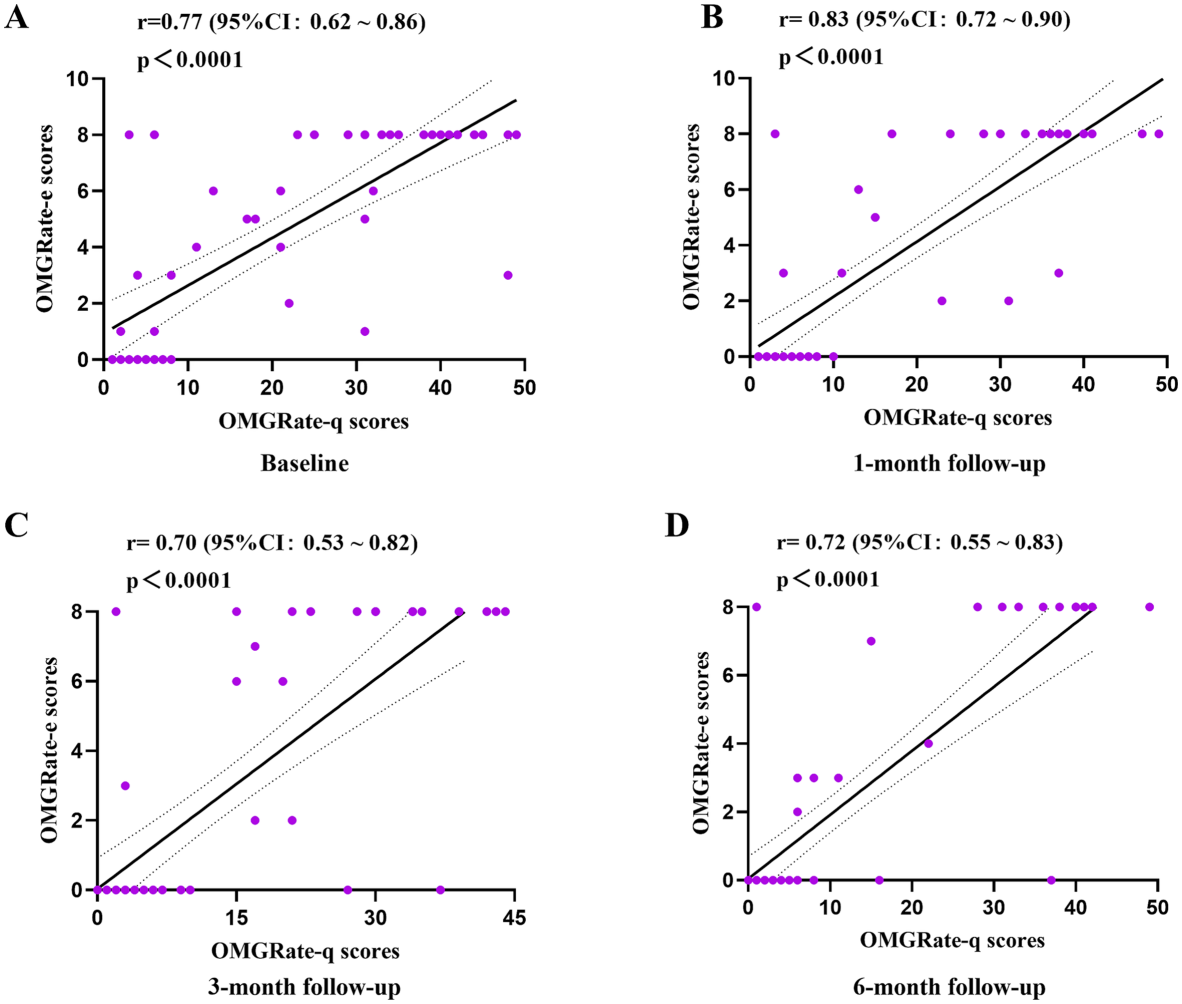
Correlation analysis between OMGRate-q and OMGRate-e. **A**, **B**, **C**, and **D** display correlations between OMGRate-e and OMGRate-q scores at baseline, 1-month, 3-month, and 6-month follow-ups, respectively. Purple dots represent individual subject scores; solid line indicates the linear regression line, and dashed lines represent the 95% confidence intervals. r: Spearman correlation coefficient, ****: *p* < 0.0001.

The correlation between OMGRate-e and OMGRate-q can be presented as evidence of convergent validity. At baseline, as illustrated in Figure 2 and Table 4, a moderate positive correlation was observed between OMGRate-e and OMGRate-q scores(*r* = 0.77, *p* < 0.0001). During the follow-up course from 1 to 6 months, OMGRate-e and OMGRate-q scores consistently demonstrated moderate (*r* = 0.70) to strong (*r* = 0.83) positive correlations (*p* < 0.0001).

**Table 4:** Dynamic correlation analysis between OMGRate and MG-QOL15, O-QMGS scale.

Scales	Baseline	1-month follow-up	3-month follow-up	6-month follow-up
OMGRate-q and OMGRate-e	0.77^****^	0.83^****^	0.70^****^	0.72^****^
OMGRate and MG-QOL15	0.39^**^	0.51^***^	0.40^**^	0.67^****^
OMGRate and O-QMGS	0.23^a^	0.43^**^	0.51^***^	0.47^***^
OMGRate-q and MG-QOL15	0.39^**^	0.49^***^	0.41^**^	0.68^****^
OMGRate-e and O-QMGS	0.21^a^	0.37^**^	0.51^***^	0.43^**^

**p<0.01, ***p<0.001, ****p<0.0001, ^a^ *p*>0.05.

### Dynamic correlation analysis between OMGRate and MG-QOL15, O-QMGS scale

Spearman correlation analysis was performed to assess the relationships among OMGRate, MG-QOL15, and O-QMGS scales before and after treatment. At baseline, as illustrated in Table 4 and Supplementary Figures S4–S7, Both the OMGRate and OMGRate-q scores showed slight positive correlations with MG-QOL15 scores (*r* = 0.39, *p* < 0.01). No other significant correlations were found among other scales at this time point (*p* > 0.05). During the follow-up course from 1 to 6 months, Mild to moderate positive correlations were identified between the following scales: OMGRate and MG-QOL15, OMGRate and O-QMGS, OMGRate-q and MG-QOL15, OMGRate-e and O-QMGS. All other inter-scale correlations remained non-significant (*p* > 0.05). By the 6-month follow-up, the correlation between OMGRate and MG-QOL15 (*r* = 0.67, *p* < 0.0001) was significantly superior to that between OMGRate and O-QMGS (*r* = 0.47, *p* < 0.001).

Collectively, these findings reveal distinct correlation patterns: a stable positive correlations were also seen between MG-QOL15 & OMGRate and MG-QOL15 & OMGRate-q. Whereas, the correlations of the O-QMGS & OMGRate and O-QMGS & OMGRate-e were only confined to the post-treatment phase. No significant association was detected between MG-QOL15 and O-QMGS.

## Discussion

Our study applied the OMGRate and MG-QOL15 to evaluate the severity of symptoms and QoL in OMG patients before and after treatment. The first validation of the collaborative value of these two scales in the Chinese OMG population, consequently, provides robust proof for clinical efficacy assessment.

Our findings demonstrate that both the age of onset and the first symptoms influence patients’ QoL. EOMG patients showed a better QoL compared to those with LOMG, while JOMG patients indicated the best QoL among the first two. This discrepancy may be caused by the fact that JOMG is confined to ocular manifestations (6), and some cases may even achieve spontaneous remission (19, 20); conversely, LOMG is associated with age-related comorbiditie (21), with a higher likelihood of psychological distress (22), which can then influence their overall health (21–23). In terms of the first symptoms, patients presenting with diplopia/strabismus or diplopia combined with ptosis showed more severe symptoms than those with ptosis only, which means diplopia imposes a substantially negative impact on visual function and QoL than ptosis to some extent. This may be because diplopia has a more immediate impact on daily activities (such as reading and driving) than ptosis alone, drawing attention to find symptoms and seeking medical attention earlier. Furthermore, AChR-Ab positivity and thymic abnormalities were regarded as factors associated with disease severity (23–27).

Our study also assessed the therapeutic effects on clinical symptoms and QoL in OMG patients by analyzing changes in OMGRate and MG-QOL15 scores before and after treatment. During the six-month observation period of this study, the results showed a notable reduction in OMGRate scores after treatment and reached their lowest point—optimal condition at the six-month follow-up, which implies effective control in core symptoms, including ptosis and diplopia. In other words, symptomatic relief means objective restoration of neuromuscular function, and indirect improvement of QoL is achieved by alleviating the limitations that visual impairments impose on daily activities. This correlation is robustly corroborated by the positive trend in MG-QOL15 scores reducing with the treatment course. By comprehensive assessment of symptom control and QoL, our study establishes a more complete evidence chain for the sequential therapeutic effect of “symptom improvement-functional recovery-quality of life enhancement”, hence resolving the previous overreliance on clinical indicators at the expense of subjective experience. The results illustrate that systematic and standardized therapy generates multidimensional benefits for patients, which means PROMs should be put into routine clinical assessment systems in the future.

Previous studies have verified the relevance between MG symptom mitigation and QoL improvement (13, 14). Through longitudinal assessment, our study first validates the dynamic relationship between OMGRate, which is considered an ocular muscle-specific tool, and the MG-QOL15 and O-QMGS scales in treatment progress in Chinese OMG patients, offering a more particular viewpoint for validity assessment in the OMG field. Our findings indicate that the OMGRate has distinctive advantages in assessing OMG therapeutic intervention. OMGRate-q and MG-QOL15 scores showed a synchronous reducing trend, with data dispersion narrowed after treatment, suggesting that they have good convergent validity and can effectively represent the relief of clinical symptoms in OMG patients (11, 12).

OMGRate-e showed better sensitivity in detecting therapy response than the traditional O-QMGS scale and showed better conformance with patients’ subjective feelings. O-QMGS is thought of as a typical method for estimating MG severity (16, 28–30), while its variation stayed steady in the course of follow-up in this research, indicating its inadequate sensitivity in capturing slight changes or the relatively milder symptoms in this study population, whereas OMGRate-e performed a more notable responsiveness than the O-QMGS scale by quantifying ptosis and diplopia (11). Accordingly, OMGRate may be considered as a simpler and pertinent alternative scale for those with OMG only (11, 12), because OMGRate can offer more comprehensive and sensitive metrics in the OMG therapeutic effect appraisal. However, the OMGRate score is driven by the OMGRate-q. Findings from our study indicate that symptom perceptions may be dissociated from clinical examination in Chinese OMG patients, which may reflect the magnifying disease impact on patients’ psychological feelings, or may relate to the milder disease severity in this study population (as indicated by stable and comparatively low O-QMGS scores).

The dynamic correlations analysis further verified the synthesis value of the OMGRate and MG-QOL15. The Cronbach’s *α* coefficient of OMGRate, OMGRate-e, and OMGRate-q indicate that the OMGRate scale has good internal consistency. The internal dimensions of the OMGRate subscales (OMGRate-e and OMGRate-q) exhibit moderate to high positive correlations in our study, demonstrating better construct validity, as clarified in previous findings (11). Additionally, OMGRate and OMGRate-q showed similar relevances with MG-QOL15; the correlation between OMGRate (OMGRate-q) and MG-QOL15 was only weakly related at baseline, but strengthened considerably over time. This initial weaker correlation suggests that OMGRate-q, which specifically quantifies ocular symptoms, but does not fully encompass the broader, multidimensional construct of QoL measured by MG-QOL15, particularly in those patients with lower symptom burden or is adapting to OMG. Consequently, in clinical practice, especially when symptoms are severe, relying solely on OMGRate may underestimate the psychosocial impact on the patient’s QoL. These two scales provides different but complementary constructs: OMGRate primarily captures the ocular signs and symptoms, whereas MG-QOL15 reflects the multidimensional influence of OMG on patients’ overall QoL. This finding notes that a combination of eye-specific scales and general QoL measures is helpful to conduct a comprehensive assessment of OMG patients’ conditions in clinical practice. Comparatively, OMGRate and OMGRate-e showed similar low to moderate relevances with O-QMGS merely after therapeutic intervention. The association gradually becomes obvious during the mid-to-late stages of treatment, as it is the collaborative function of clinical symptom relief and functional status restoration rendered in longitudinal validity. This phenomenon hints that OMGRate is a better tool that can simultaneously reflect clinical symptom relief and patients’ subjective feelings sensitively. The OMGRate can be used as a bridge to connect impersonal signs and subjective experiences, then and address deficiencies in existing assessment systems. Therefore, we advocate for the combined use of OMGRate and MG-QOL15; this dual-scale approach is crucial for nuanced, patient-centered care and shared decision-making, especially when symptoms are severe.

In conclusion, by integrating the OMGRate and MG-QOL15 assessment methods, our study verifies the dual benefits of personalised treatment strategies in enhancing clinical symptoms and QoL in OMG patients. Our study provides cross-cultural validation of the internal consistency and reliability of the OMGRate scale in the Chinese population and offers preliminary validation of the OMGRate as an effective method in disease severity evaluation in Chinese OMG patients, providing foundational data for future research. The findings demonstrate that OMG severely impairs patients’ QoL, and that appropriate intervention therapy can effectively alleviate clinical symptoms and improve QoL simultaneously, and these two dimensions show good consistency.

This study has several limitations. First, no official Chinese version is available currently, which is a significant limitation of this research. Second, we did not systematically exclude non-adherent patients, which may introduce variability in treatment exposure; it is impossible to distinguish between therapeutic effects and placebo effects or the natural course of the disease. Third, as a single-center study with a small sample size, a small sample size resulted in a wide CI, leading to insufficient power for some subgroup analyses, and a selection bias limits the generalizability of the findings. Fourth, this study did not systematically collect or adjust for comorbidities such as cardiovascular disease. Consequently, this association cannot be directly attributed to MG itself for the confounded disease. Last but not least, the relatively short follow-up period may have limited a comprehensive evaluation of the long-term treatment efficacy on QoL of its change trend and the potential risk of disease conversion.

Based on the limitations of this study, we recommend that future research aiming to develop a standardised Chinese version of OMGRate. Subsequent research should involve multicenter medical centers, expand sample sizes, set appropriate control groups, and extend follow-up durations to validate our conclusions and to explore the longitudinal patterns of change of QoL and clinical indicators across different treatment phases. Moreover, this study’s primary reliance on PROM may introduce recall bias and social expectation bias. More objective assessment methods should be introduced to improve data accuracy in the future.

## Conclusion

In summary, this study confirms the combined application value of the OMGRate and MG-QOL15. They can evaluate symptomatic improvement and changes in QoL comprehensively among OMG patients, providing proof for clinical decision-making. Promoting OMGRate holds significant importance for prognostic evaluation in clinical practice. Future efforts should focus on carrying out multicenter, large-sample studies to validate the universality of the scale. Furthermore, leveraging artificial intelligence and digital assessment methods can enhance the objectivity of disease monitoring, optimize remote management for OMG, and promote the development of precise and individualized treatment strategies.

## Data Availability

The datasets presented in this article are not publicly available due to privacy or ethical restrictions but are are available from the corresponding author upon reasonable request. Requests to access the datasets should be directed to QZ, kerryzh@163.com.

## Supplementary material

Supplementary FIGURE S1 Dynamic changes in OMGRate, MG-QOL15, and O-QMGS scores before and after therapeutic intervention (**p* < 0.05, ***p* < 0.01, ****p* < 0.001,*****p* < 0.0001).

Supplementary FIGURE S2 Distribution of OMGRate-e scores.

Supplementary FIGURE S3 Distribution of O-QMGs scores.

Supplementary FIGURE S4 Correlation analysis between OMGRate and MG-QOL15.

Supplementary FIGURE S5 Correlation analysis between OMGRate and O-QMGS.

Supplementary FIGURE S6 Correlation analysis between OMGRate-q and MG-QOL15.

Supplementary FIGURE S7 Correlation analysis between OMGRate-e and O-QMGS.
