# Supplementary figures and images for: Validation and clinical utility of OMGRate and MG-QOL15 in evaluating symptoms and quality of life in Chinese patients with ocular myasthenia gravis
Source: Front Neurol. 2026 May 22;17:1842388. doi: 10.3389/fneur.2026.1842388 (PMC13238584; doi:10.3389/fneur.2026.1842388)

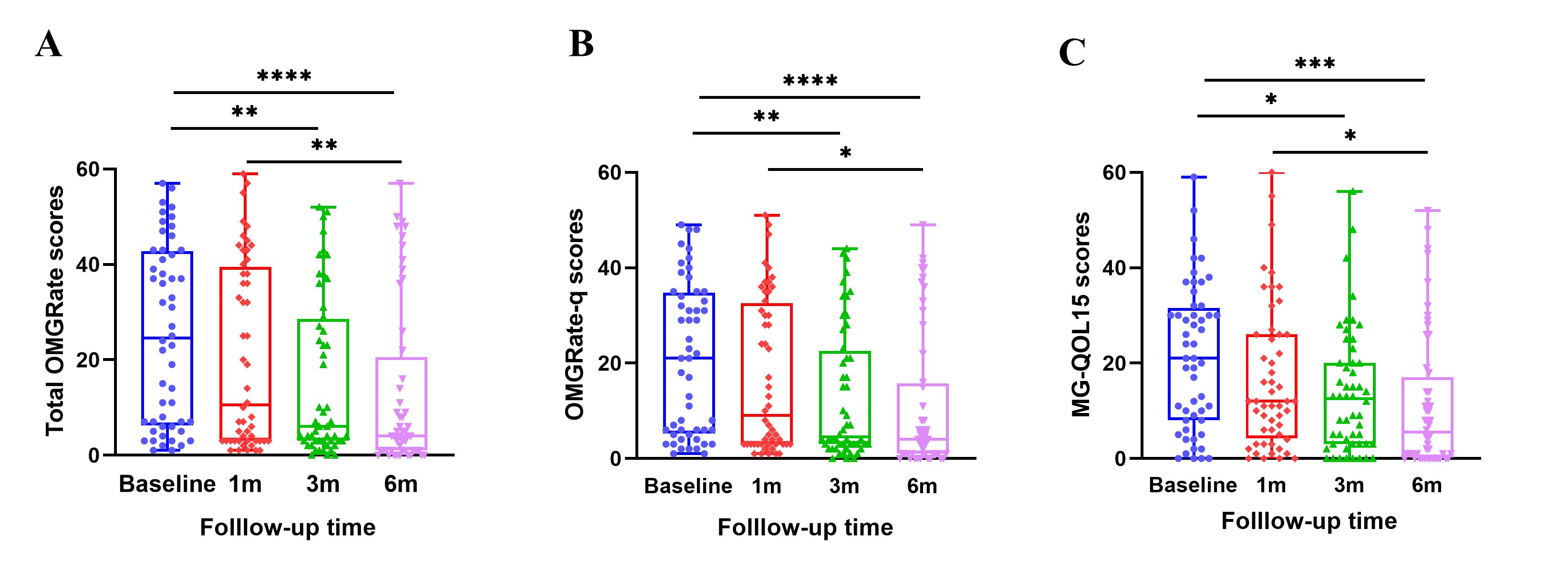

Supplement: Supplementary FIGURE S1 — Dynamic changes in OMGRate, MG-QOL15, and O-QMGS scores before and after therapeutic intervention (*p < 0.05, **p < 0.01, ***p < 0.001,****p < 0.0001). [file Image_1.TIF]

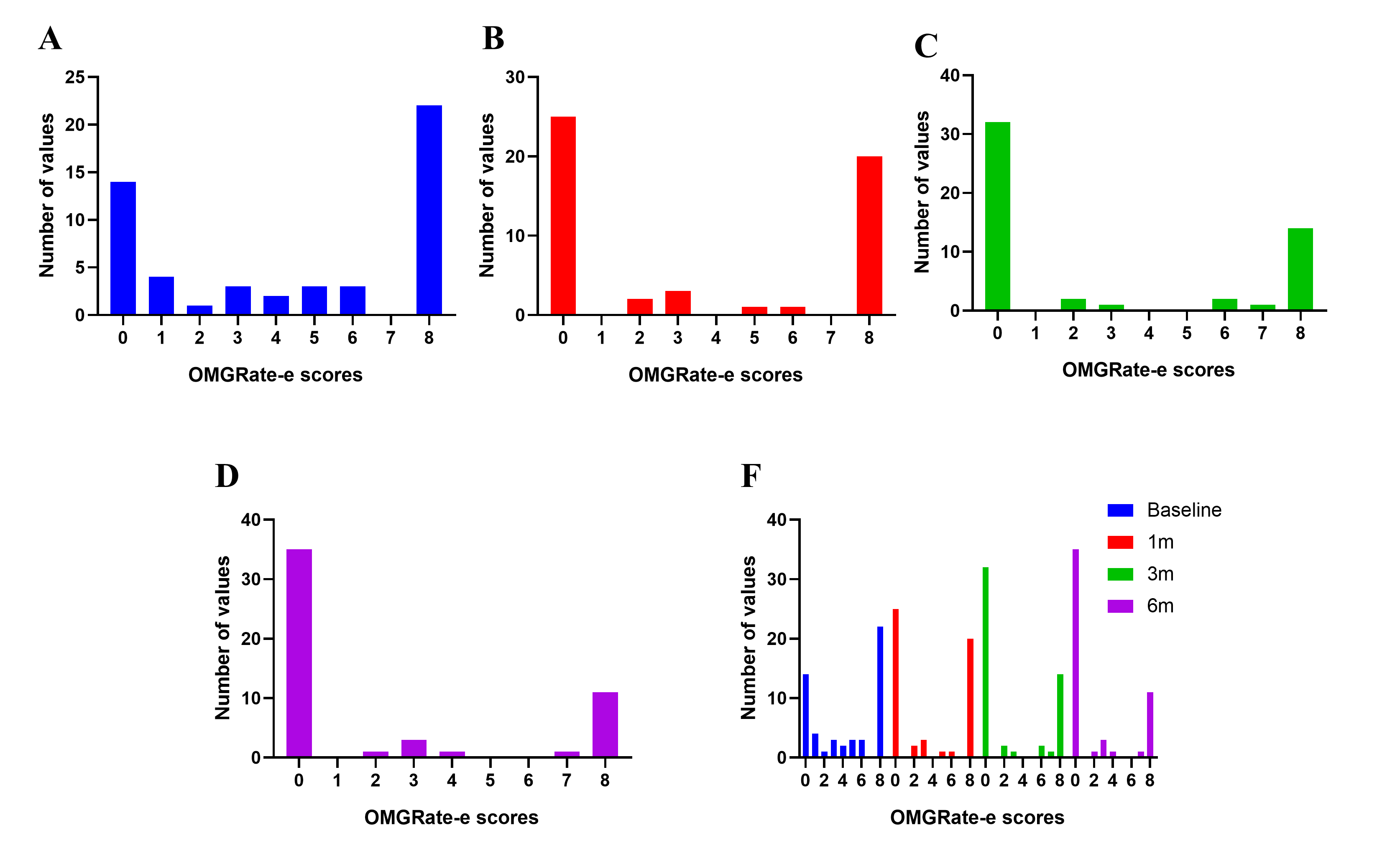

Supplement: Supplementary FIGURE S2 — Distribution of OMGRate-e scores. [file Image_2.TIF]

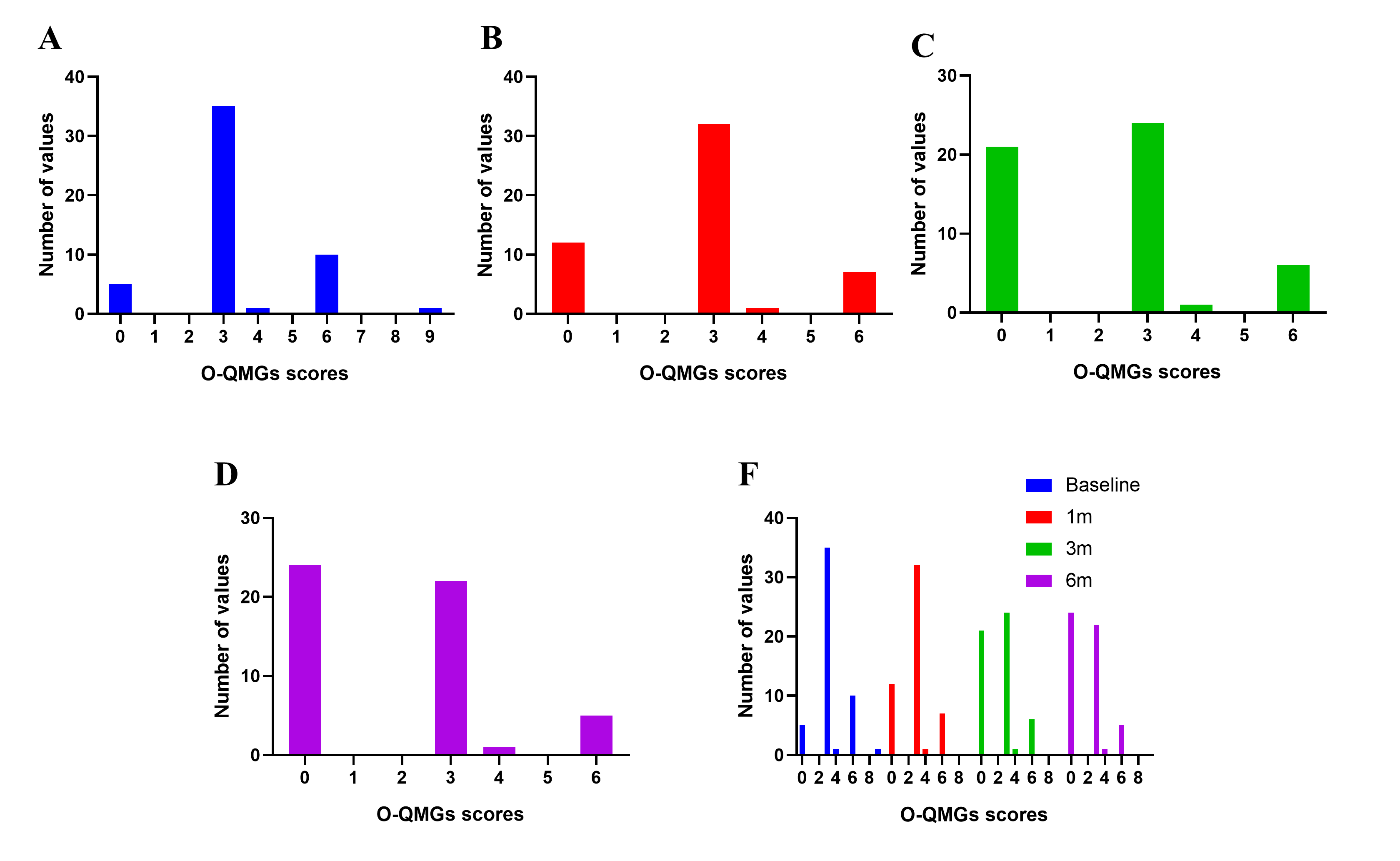

Supplement: Supplementary FIGURE S3 — Distribution of O-QMGs scores. [file Image_3.TIF]

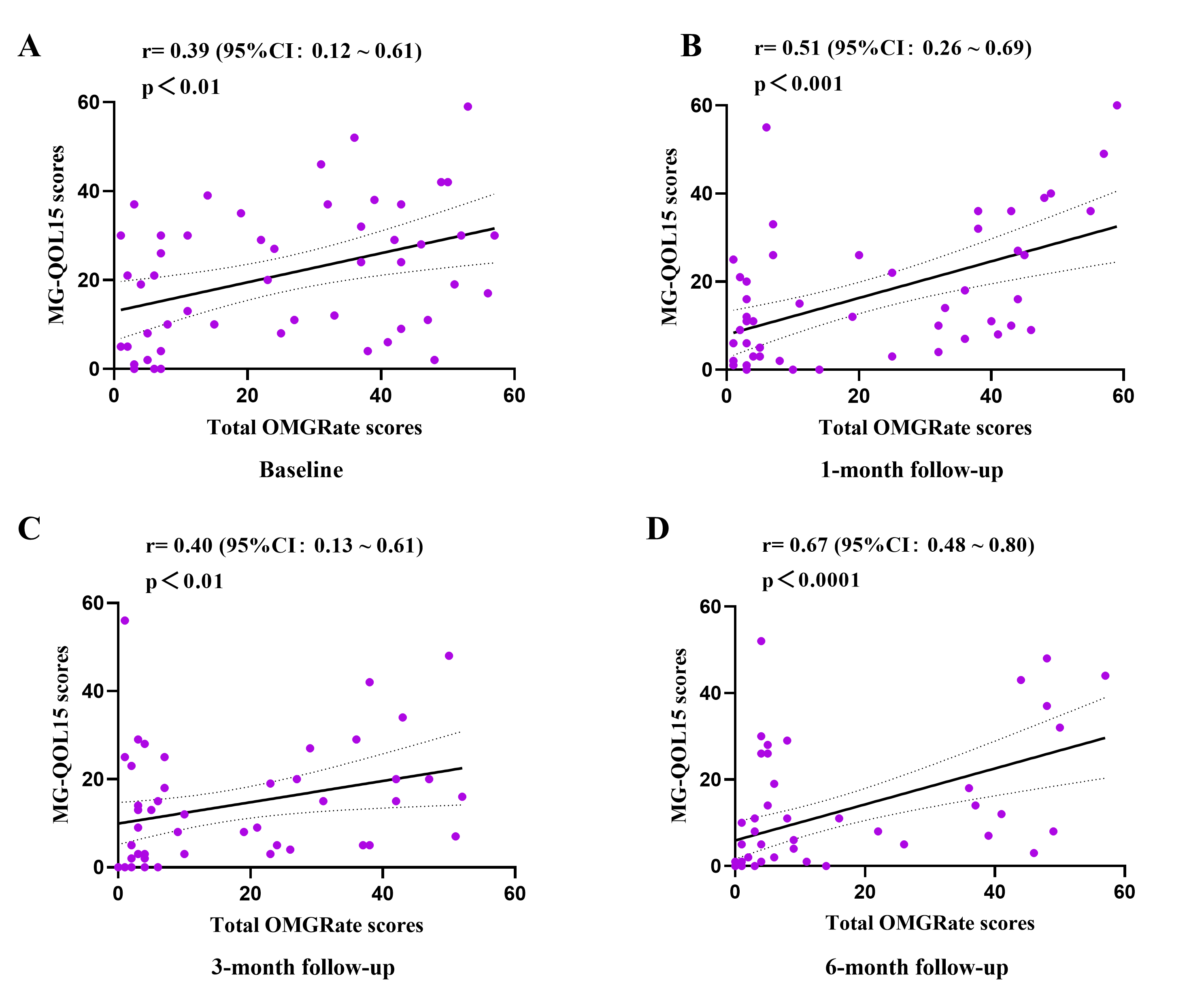

Supplement: Supplementary FIGURE S4 — Correlation analysis between OMGRate and MG-QOL15. [file Image_4.TIF]

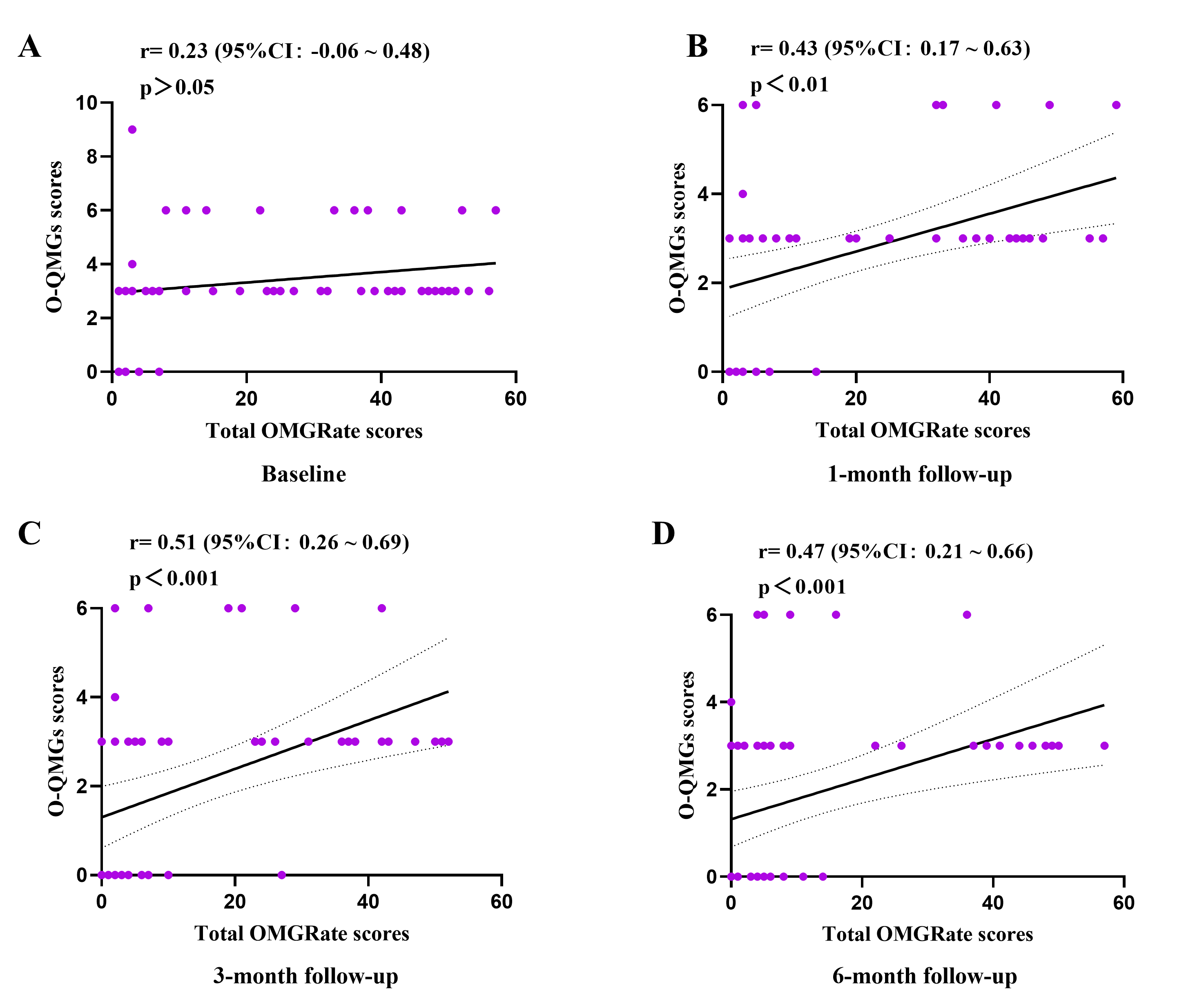

Supplement: Supplementary FIGURE S5 — Correlation analysis between OMGRate and O-QMGS. [file Image_5.TIF]

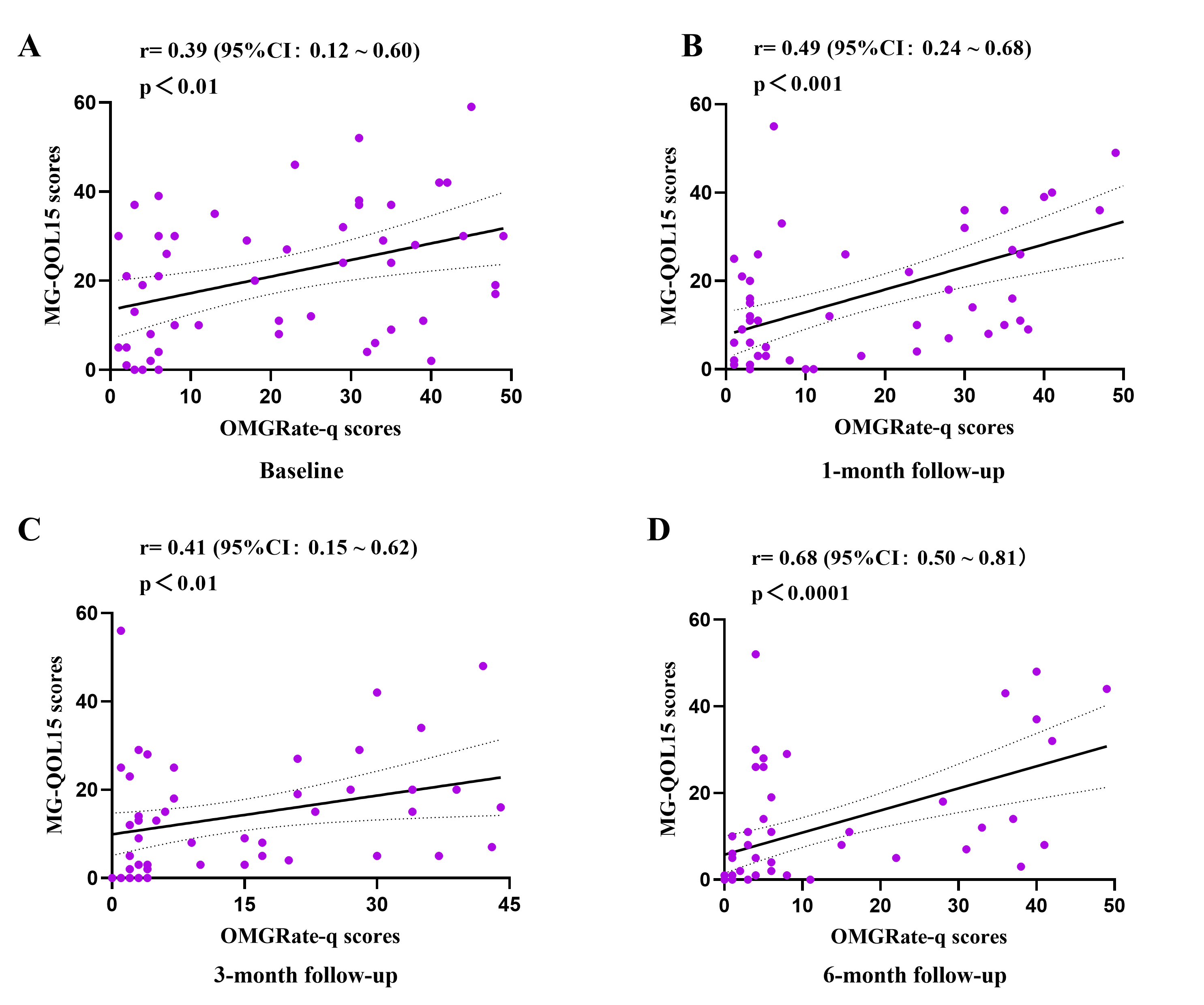

Supplement: Supplementary FIGURE S6 — Correlation analysis between OMGRate-q and MG-QOL15. [file Image_6.TIF]

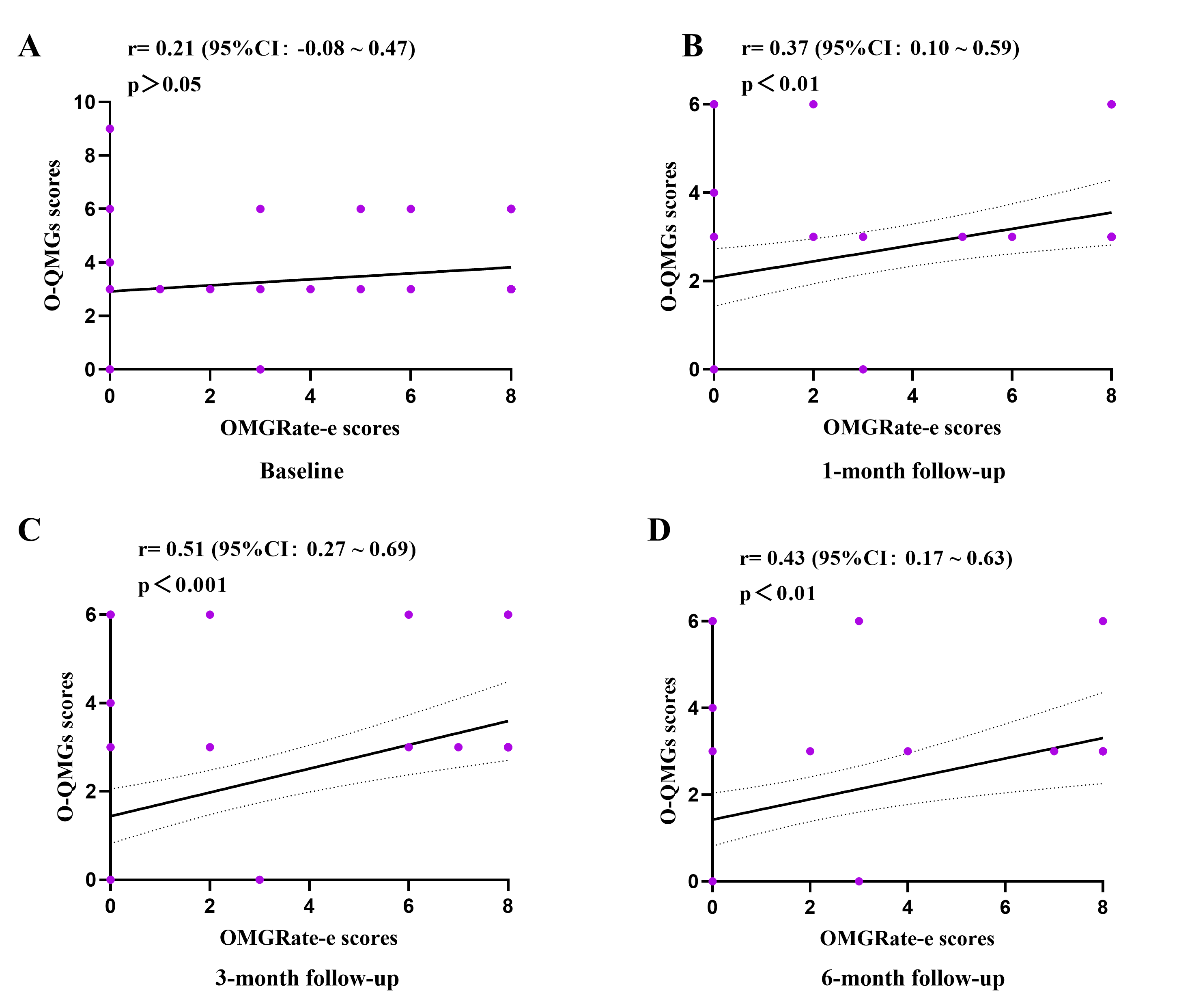

Supplement: Supplementary FIGURE S7 — Correlation analysis between OMGRate-e and O-QMGS. [file Image_7.TIF]
