# Supplementary material for: Validation and clinical utility of OMGRate and MG-QOL15 in evaluating symptoms and quality of life in Chinese patients with ocular myasthenia gravis
Source: Front Neurol. 2026 May 22;17:1842388. doi: 10.3389/fneur.2026.1842388 (PMC13238584; doi:10.3389/fneur.2026.1842388)
Supplement: Supplementary file 8 [file Table_1.docx]

Table S1 Participant Demographics and Characteristics

| Characteristic | Category | Number (n) | Percentage ( %) |
| --- | --- | --- | --- |
| Sex | Male | 19 | 36.5 |
|  | Female | 33 | 63.5 |
| Age of Onset | JOMG | 13 | 25 |
|  | EOMG | 28 | 53.8 |
|  | LOMG | 11 | 21.2 |
|  | Mean ± SD (34.0± 19.1) |  |  |
| First Symptoms | Ptosis only | 12 | 23.2 |
|  | Diplopia / Strabismus | 25 | 48.1 |
|  | Ptosis with Diplopia / Strabismus | 15 | 28.8 |
| Ice Pack Test | Positive | 35 | 79.5 |
|  | Negative | 9 | 20.5 |
| Serum AChR-Ab Test | Positive | 6 | 12.5 |
|  | Negative | 42 ^#^ | 87.5 |
| RNS | Positive | 3 | 12.5 |
|  | Negative | 21 | 87.5 |
| Neostigmine Test | Positive | 36 | 80 |
|  | Negative | 9 | 20 |
| Thyroid | Abnormal | 31 | 64.6 |
|  | Normal | 17 | 35.4 |
| Thymus | Abnormal | 21 | 42.9 |
|  | Hyperplasia/Incomplete involution | 20 | 40.8 |
|  | Thymoma | 1 | 2.1 |
|  | Normal | 28 | 57.1 |
| Immune function | Disorder | 31 | 68.9 |
|  | Normal | 14 | 31.1 |
| Therapeutic strategy | Acetylcholinesterase inhibitors | 34 | 65.38 |
|  | Acetylcholinesterase inhibitors + immunosuppressive drugs | 18 | 34.62 |

# Among the AChR-Ab negative patients, the diagnosis of OMG was confirmed by RNS in 3 patients (7.14%), positive neostigmine test in 36 patients (85.72%), and symptoms significantly relieved after treatment in 3 patient (7.14%).
